# The potential impact of introducing a cost tool to facilitate cost-of-care conversations in routine OB care: Lessons from the CONTINUE pilot study

**DOI:** 10.1016/j.pecinn.2023.100136

**Published:** 2023-02-15

**Authors:** Veronica Fitzpatrick, Kim Erwin, Anne Rivelli, Maureen Shields, Leah Delfinado, Marie Cabiya, Karen Wennerberg

**Affiliations:** a Advocate Aurora Research Institute, Downers Grove, IL, USA; b Advocate Aurora Health; Downers Grove, IL, Milwaukee, WI, USA; c Illinois Institute of Technology Institute of Design, Chicago, IL, USA

**Keywords:** Cost-of-Care Conversations, Maternal Child Health, Vulnerable Populations, OB

## Abstract

**Objective:**

The objective of the CONTINUE study is to gather preliminary data on the potential impact of implementing a “Cost Tool” in routine obstetrics (OB) care. It is hypothesized that by providing prenatal patients with an ability to forecast their care plan, they would be better able to anticipate and plan for the costs associated with their prenatal care.

**Methods:**

Pilot data from interviews and surveys were collected from 71 prenatal patients across three clinics throughout Chicago, IL.

**Results:**

As compared to privately insured prenatal patients, prenatal patients with public insurance reported the most benefit in Cost Tool use. Specifically, that the Cost Tool helped to navigate insurance more effectively (OR 4.49, p=0.0254), see the "Big Picture" and link it to the family budget (OR 4.25, p=0.0099), and make the financial tradeoffs needed to get through pregnancy (OR 5.50, p=0.0305).

**Conclusion:**

The CONTINUE study provides preliminary signals of the Cost Tool’s potential to help publicly insured prenatal patients better navigate the costs associated with their care plan.

**Innovations:**

The CONTINUE study contributes valuable preliminary data about the utility of a cost tool in routine OB care, especially as it may benefit low-income prenatal patients navigate prenatal care better.

## Highlights

• low-income prenatal patients benefited from using well-designed validated tool.
• Low-income prenatal patients welcome less cost conversations with providers.
• More research should be conducted to capture the effects of tools in OB care.

## 1 Introduction

Conversations about costs (“cost-of-care conversations”) can lead to better health care outcomes by improving patient adherence to care plans and engagement in the management of their care [1,2]. However, little exists to help clinicians understand how best to engage in cost-of-care conversations, despite patient-provider cost-of-care conversations becoming increasingly viewed as an integral part of delivering high-value care [1,2]. Cost-of-care conversations can enhance clinical decision-making and lead to reduction in risk of financial hardship by prompting an evaluation of medical-financial tradeoffs, thus increasing care plan adherence [1,3].

Studies suggest that more than half of patients want to engage in cost-of-care conversations and for their medical provider to factor costs into their care plans [[4], [5], [6], [7]]. Yet, fewer than 1 in 5 patients report having cost discussions with their medical providers [8]. Many physicians report initiating cost-of-care conversations only when they sense patients are having financial difficulties; however, a study found that physicians were prone to missing patient cues about financial burden, and evidence indicates that physicians are underprepared to address these cues even when recognized [9,10]. Despite research showing that both clinicians and patients want to engage in cost-of-care conversations, conversations are still not occurring as frequently or effectively as they could [1,11,12]. Cost-of-care conversations are particularly important to vulnerable patients – low-income and/or patients with high-cost conditions such as cancer, psychiatry, and pregnancy – who are more likely to delay or forgo needed care (such as doctor visits, treatments, and medications) due to cost [4].

A supplement published by *Annals of Internal Medicine* in 2019 focused on cost-of-care conversations and how to improve them. Within this, several provider barriers to effective cost-of-care conversations were identified, which included: insufficient time and expertise, lack of awareness of available resources, lack of knowledge about the costs of specific tests and treatments, difficulty estimating the total costs of a care plan, difficulty estimating nonmedical (indirect) costs associated with care, and discomfort initiating and conducting conversations about costs-of-care [[13], [14], [15], [16]]. A 2017 literature review found that, although 75% of surveyed physicians felt that initiating cost-of-care conversations was their responsibility, less than 30% felt comfortable introducing the conversations [5]. More recently, a 2020 systematic review corroborated earlier research stating that, although there is a desire for these conversations to take place, there is a lack of evidence-based guidelines for encouraging cost conversations and improving their quality [4].

To support physicians and patients with integrating cost-of-care conversations into routine clinical practice, several healthcare stakeholder organizations, including the American College of Physicians (ACP), American’s Essential Hospitals, and Robert Wood Johnson Foundation, are working to standardize these conversations and promote their usage in routine clinical care. Given the research above, it is generally agreed upon that these conversations need to happen systematically and with all patients, irrespective of condition and perceived financial status. Additionally, recent interventions and research around the efficacy of cost-of-care conversations have concluded that these conversations are better facilitated when supplemented with conversational aids such as sample scripts, tools, handouts, and electronic medical record (EMR) reminders [13,17]. The CONTINUE study fills an important research gap by piloting a cost-of-care tool (“Cost Tool”) in routine obstetrics (OB) care to gather preliminary data on patient experiences with the Cost Tool on cost-of-care conversations and additional cost-related benefits. This study hopes to further research in this important area.

### 1.1 CONTINUE study

The CONTINUE (**Co**st Co**n**versa**ti**ons i**n** Ro**u**tine OB Car**e**) study piloted and documented the potential impact of implementing the Cost Tool in routine OB care with prenatal patients over a one-year time period in multiple clinics located throughout Chicago, IL. Pregnancy care plans are inherently burdensome and expensive. Typical prenatal care can require up to 42 weeks of visits to OB providers, including labs, monitoring, and other standard-of-care evaluations, all of which have associated direct and indirect – not insurance related – costs. While prenatal care is burdensome to most pregnant people, particularly those with high-risk pregnancies, low-income prenatal patients, regardless of risk status, are more likely to defer, decline, or engage poorly in pregnancy care due to cost [18,19]. In addition, low-income prenatal patients express significantly more dissatisfaction than middle-income patients with the availability of healthcare cost information [20].

**Fig. 1:**
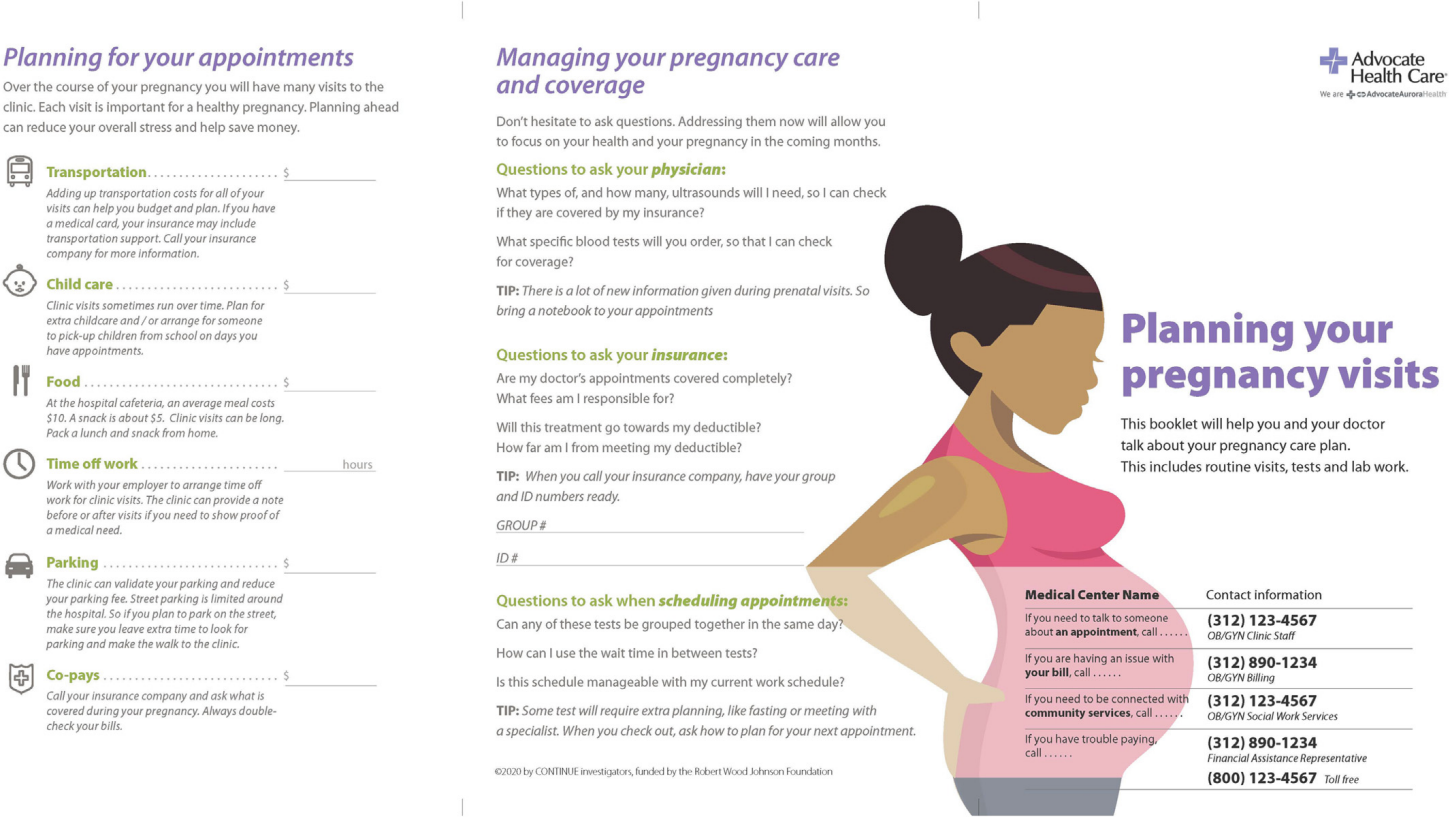

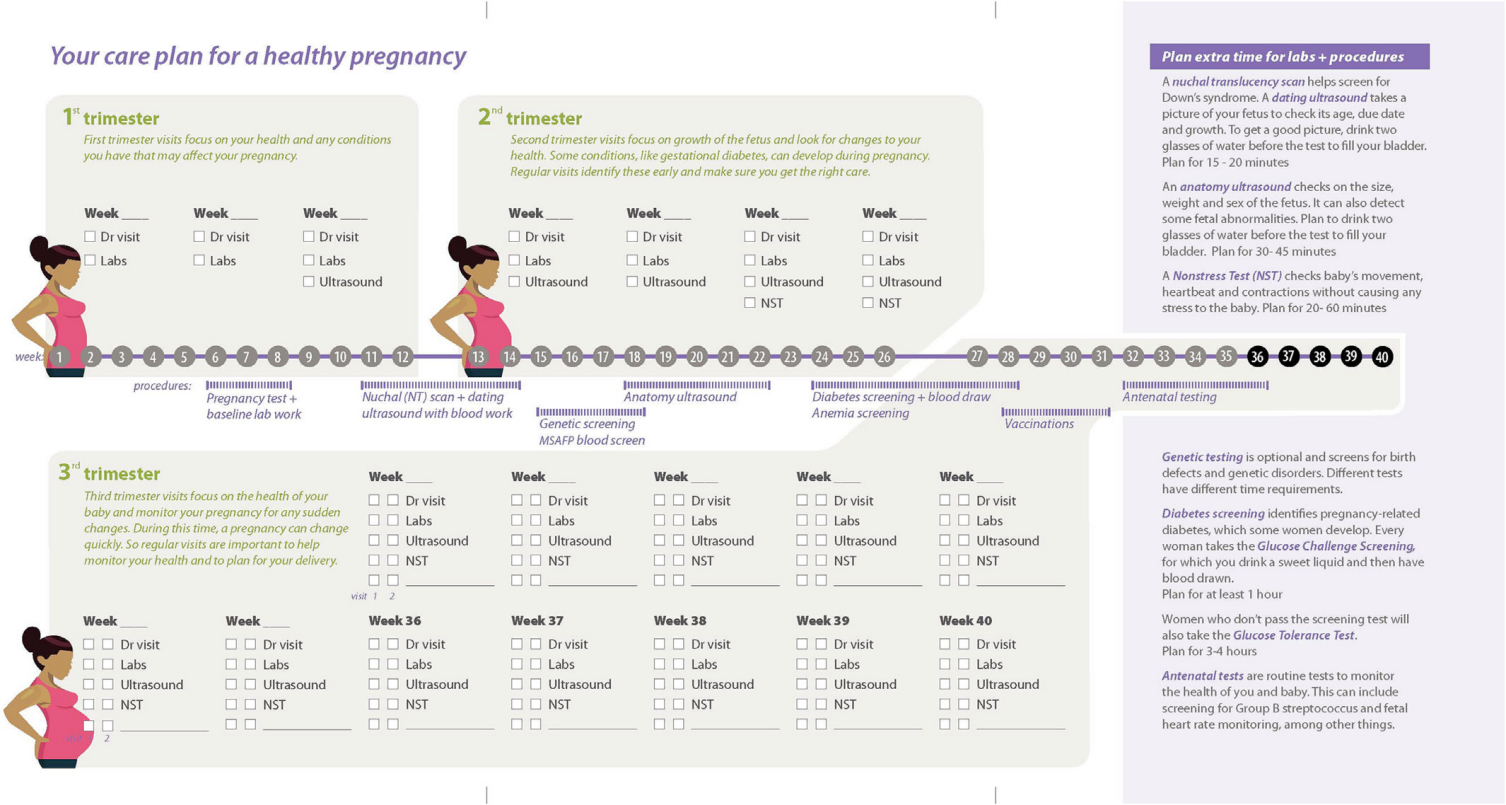
Cost-of-Care Tool

Previous research suggests that the indirect costs of prenatal care, driven by frequency and duration of appointments, are the most substantial financial burden for low-income prenatal patients. Guideline-based prenatal care is visit-intensive, requiring 12 to 27 visits for low and high-risk pregnancies, respectively [21]. Additionally, prenatal patients and clinic staff report that clinic visits, especially third-trimester visits that involve testing and fetal monitoring, can routinely last up to 4 hours. Cumulatively, even when care is “free” because of Medicaid coverage, indirect costs can exceed $2500 during high-risk prenatal care [22]. Indirect costs most often identified as barriers to care include: transportation to and from appointments, parking costs, childcare, and wage loss [22]. Previous research hypothesizes that clinic-based conversations about indirect costs-of-care could help prenatal patients anticipate and plan for these costs, subsequently improving patient adherence to care plans and restoring the interpersonal aspects of prenatal care—patient trust, partnership, and a sense of shared purpose with clinicians—that suffer when patients fail to adhere to care plans. While many pregnancy support tools have been created, few have been sustained and integrated into routine OB care [23]. The primary objective of the CONTINUE study is to gather preliminary data from the integration of a validated Cost Tool (Fig. 1) on prenatal patients in multiple real-world clinical OB settings.

### 1.2 The Cost Tool

The Cost Tool is a paper-based tool that was iteratively co-designed with low-income prenatal patients, physicians, and nurses to function as a “boundary object;” a shared informational tool used to bridge and mediate the communication between stakeholder groups with different knowledge levels and goals. A key function of boundary objects is to provide a shared language that is meaningful to all stakeholders, which includes clear, recognizable content and framing [24]. Further, paper-based tools have been shown to improve shared review of information and to increase eye contact between the patient and provider compared to digital media [25]. Tool development involved a multistakeholder engagement process generating thirteen tool iterations that resulted in an optimized paper-based tool that allows providers and prenatal patients to discuss the entirety of the care trajectory^.^ The final Cost Tool integrates appointments, milestones and diagnostics into a comprehensive action plan, using familiar OB pregnancy constructs and checkboxes to align it with OB workflow and reduce completion time to one minute or less.

## 2 Methods

To gather pilot data on the impact of integrating a validated Cost Tool into routine OB care, the CONTINUE study enrolled prenatal patients from three outpatient OB clinics served by four different OB provider types. OB providers were instructed to give the Cost Tool to “New OB” patients (a clinical denotation in the electronic medical record (EMR) identifying patients presenting for their first prenatal care appointment for the current pregnancy episode) during a 14-week pilot. The Cost Tool was available in both English and Spanish. Each 14-week pilot was held sequentially and conducted between September 2020 and July 2021. Directly prior to tool implementation, most OB providers underwent a one-hour in-person training. Training consisted of three components: 1) Tool usage: filling out the tool correctly and distributing it with fidelity, 2) Implementation logistics: identifying where tools will be placed in the clinic to maximize usage (e.g., exam rooms, procedure rooms, next to the computer) and where in the electronic medical record (EMR) to track tool distribution, and 3) Site readiness: assessing local barriers or preparatory activities to be addressed by the local team. At the end of the training event, providers received an unlimited number of pregnancy support tools for distribution in their clinic. Pregnancy support tools were pre-labeled with unique tracking numbers on each tool, reflecting sequential order and clinic (e.g. ASMC102).

Data was collected between January 2021 and December 2021. A mixed-methods approach to data collection was used in the forms of interviews and surveys. This study was approved by Advocate Health Care IRB (#20-264E).

### 2.1 Clinic and patient demographics

The CONTINUE study team targeted clinics with different OB provider types – attending OB, resident OB, midwives, and nurses. Clinic 1 serves mid to higher-income, privately insured, predominantly White, high- and low-risk patients, with four attending OB providers on staff. Clinic 2 serves low-income, publicly insured, predominantly Hispanic, high- and low-risk patients. Clinic 2 is served by 12 resident OB providers, each rotating for four weeks, and three nurse providers on staff. Clinic 3 serves higher-income, privately insured, predominantly White, low-risk patients, with two midwife providers on staff.

Patients were given the Cost Tool by their OB provider at their New OB appointment (typically 8-12 weeks pregnant). All New OB patients were eligible for the Cost Tool. To be eligible for follow-up, prenatal patients had to be at least 18 years old, have used the Cost Tool for at least 8 weeks, and be at least 27 weeks pregnant. Additionally, demographics and care-related data were collected during interviews and surveys. Table 1 describes participant demographics.

**Table 1:** Patient demographics (N = 71).

Age Group	
18-25	12 (18.3%)
26-35	41 (57.8%)
36-45	17 (23.9%)
Race	
AI/AN	1 (1.4%)
Asian	4 (5.7%)
Black	9 (12.9%)
Other	6 (8.6%)
White	50 (71.4%)
Ethnicity	
Hispanic/Latino/Spanish	40 (56.3%)
Non-Hispanic/Latino/Spanish	31 (43.7%)
Language	
English	68 (95.8%)
Spanish	3 (4.2%)
Insurance⁎ Statistically significant at p<0.05	
Public (Low)	40 (56.3%)
Private (Higher)	31 (43.7%)
Provider Type	
Midwife	12 (16.9%)
Nurse	17 (23.9%)
OB	19 (26.8%)
Resident	23 (32.4%)

⁎ Insurance is being used as a proxy for income in this study and will be referred to as insurance throughout this paper.* See Variables section.

To capture experiences with the Cost Tool, prenatal patients were offered either an interview or survey after their 27^th^ week of pregnancy. Prenatal patient participants self-selected to complete a semi-structured interview or a surrogate electronic survey. The interview was based on human-centered design (HCD) methodologies which were employed during the tool development phase [22] and adapted for the CONTINUE pilot. Interviews were led by two research team members and prenatal patients were asked to pull pictures of emotions they related to, journey map the costs incurred during their pregnancy, and card sort their experiences with the Cost Tool into what they did or did not experience. Survey participants reviewed the same card sort items as the interviewees in the electronic survey, with each item as a unique survey question. Although self-selection may introduce bias into the results, due to COVID-19 limitations at the time of the study, in-person interviewing was not always acceptable, and few people had a video conferencing ability. The interviews and the surveys covered the same items.

### 2.2 Cost-of-Care measures

The CONTINUE study assessed both who cost-of-care conversations were (or were not) occurring with and what additional financially-related benefits the implementation of the Cost Tool into routine OB care may have. Cost-of-care implementation measures for the CONTINUE study were formulated with prenatal patients and OB providers in a prior study [22]. A total of 18 Cost Tool benefits were identified; five items addressed financial issues related to prenatal care. At launch, the CONTINUE study confirmed all five financial items (Table 2).

**Table 2:** Cost measure survey questions.

Cost-of-Care Conversation Items
(1) Did cost conversations occur with provider?
(2) Did cost conversations occur with partner?
(3) Did cost conversations occur with family member(s)?
(4) Did cost conversations occur with friend or coworker?
(5) Did cost conversations occur with someone at clinic (not provider)?
(6) Did cost conversations occur with insurance provider?
(7) Did cost conversations occur with HR/benefits specialist at work?
(8) Do you believe that incorporating cost-related conversations into pregnancy care is important?
Cost Tool Implementation Benefit Outcome Items
(1) The cost tool helped me navigate insurance more effectively.
(2) The cost tool helped me see the "Big Picture" and link it to the family budget.
(3) The cost tool helped me feel my financial situation was being considered.
(4) The cost tool helped me make the financial tradeoffs needed to get through my pregnancy.
(5) The cost tool helped me manage my other life responsibilities.

### 2.3 Variables

Demographic variables were collected on all prenatal patients, including: Age, categorized, 18-25, 26-35, 36-45; Race, White, Black, Asian, AI/AN, and other; Ethnicity, Hispanic/Latino/Spanish and Non-Hispanic/Latino/Spanish; Insurance status, public and private; Language, English and Spanish; Currently working, yes or no; and Current partner; yes or no. Pregnancy specific variables were also collected, including: Provider type, midwife, nurse, OB, attending; Parity, first or subsequent; Risk, high, low, or unknown; and Clinic, 1, 2 or 3. It is important to note that this study aimed to identify differences in the cost measure by income status; however, to prevent prenatal patients from feeling targeted based on perceived income level, income was not asked in data collection. Instead, insurance status is being used as a proxy for income in this study, with publicly insured prenatal patient participants defined as low-income and privately insured prenatal patient participants defined as higher-income and will be referred to as insurance status (public vs. private) throughout this paper.

In total, the cost measure included eight cost-of-care conversation outcome items and five Cost Tool implementation benefit outcome items and was collected using interviews and surveys. Items from the semi-structured interview card sort activity directly corresponded to items from the electronic survey, with each item as a unique statement, and therefore for this manuscript, all cost-related items are treated as quantitative. See Table 2 for complete list of items.

### 2.4 Statistical methodology

Data management and analysis were performed by the study research team using SAS statistical software (Version 9.4; SAS Institute, Cary, NC). Descriptive statistics are reported as counts (%). Univariate and bivariate analyses were conducted to examine the distributions of each variable, among the overall sample and by insurance type. Reported measures of association include odds ratios (OR) with associated 95% confidence intervals (CI). Because this study hypothesized a greater impact of the pilot Cost Tool on low-income prenatal patient participants and public insurance was used as the proxy for low-income, ORs are interpreted from the perspective of prenatal patients with public insurance. Specifically, in Table 3, OR describe the odds of sample prenatal patients of a given variable level (relative to the identified reference level) having public insurance relative to private insurance. Corresponding p-values were generated from Chi square tests (or Fishers Exact Tests if any cell counts were less than 5) to represent statistically significant differences in distributions of baseline demographics- and care-related variables by insurance type. In Table 4, cost measure ORs describe the odds of experiencing cost conversations or Cost Tool benefits among prenatal patients with public insurance relative to those with private insurance. Corresponding p-values were generated from Chi square tests (or Fishers Exact Tests if any cell counts were less than 5) to represent statistically significant differences in experiencing cost-related outcomes. Alpha of p<0.05 was used to determine statistical significance in all analyses.

**Table 3:** Sample demographic- and care-related variables, by insurance status.

Overall (N=71)		Insurance			
		Public (N=40)	Private (N=31)	OR^^^	P-Value^+^
Data Type					
Interview	37 (52.1%)	19 (51.4%)	18 (48.7%)	0.65 (0.25, 1.68)	0.3768
Survey	34 (47.9%)	21 (61.8%)	13 (38.2%)	REF	
Language					
English	68 (95.8%)	37 (54.4%)	31 (45.6%)	-	0.2515^
Spanish	3 (4.2%)	3 (100.0%)	0 (0.0%)	-	
Race					
AI/AN	1 (1.4%)	1 (100.0%)	0 (0.0%)	-	0.3343^
Asian	4 (5.7%)	1 (25.0%)	3 (75.0%)	0.31 (0.03, 3.16)	
Black	9 (12.9%)	7 (77.8%)	2 (22.2%)	3.23 (0.61, 17.10)	
Other	6 (8.6%)	4 (66.7%)	2 (33.3%)	1.85 (0.31, 11.01)	
White	50 (71.4%)	26 (52.0%)	24 (48.0%)	REF	
Ethnicity					
Hisp/Lat/Span	40 (56.3%)	30 (75.0%)	10 (25.0%)	6.30 (2.23, 17.80)	0.0003⁎ Statistically significant at p<0.05
Non-Hisp/Lat/Span	31 (43.7%)	10 (32.4%)	21 (67.7%)	REF	
Clinic					
Clinic 1	19 (26.8%)	1 (5.3%)	18 (94.7%)	REF	<0.0001^⁎ Statistically significant at p<0.05
Clinic 2	40 (56.3%)	36 (90.0%)	4 (10.0%)	162.00 (16.85, >999.999)	
Clinic 3	12 (16.9%)	3 (25.0%)	9 (75.0%)	6.00 (0.54, 66.17)	
Provider Type					
Midwife	12 (16.9%)	3 (25.0%)	9 (75.0%)	6.00 (0.54, 66.17)	<0.0001^⁎ Statistically significant at p<0.05
Nurse	17 (23.9%)	13 (76.5%)	4 (23.5%)	58.50 (5.84, 586.123)	
OB	19 (26.8%)	1 (5.2%)	18 (94.7%)	REF	
Resident	23 (32.4%)	23 (100.0%)	0 (0.0%)	-	
Age Group					
18-25	12 (18.3%)	12 (92.3%)	1 (7.7%)	8.50 (1.01, 71.70)	0.0006^⁎ Statistically significant at p<0.05
26-35	41 (57.8%)	24 (58.5%)	17 (41.5%)	REF	
36-45	17 (23.9%)	4 (23.5%)	13 (76.5%)	0.22 (0.06, 0.79)	
Partner (N=37)					
Yes	36 (97.3%)	0 (0.0%)	1 (100.0%)	-	1.0000^
No	1 (2.7%)	18 (50.0%)	18 (50.0%)	-	
Risk (N=67)					
HR	28 (39.4%)	17 (60.7%)	11 (39.3%)	1.19 (0.44, 3.21)	0.7246
LR	39 (54.9%)	22 (56.4%)	17 (43.6%)	REF	
Unknown^#^	4 (5.6%)	1 (25.0%)	3 (75.0%)	-	
Parity (N=70)					
First	30 (42.3%)	16 (53.3%)	14 (46.7%)	0.85 (0.33, 2.19)	0.7284
Subsequent	40 (56.3%)	23 (57.5%)	17 (42.5%)	REF	
Work (N=36)					
Yes	24 (66.7%)	8 (33.3%)	16 (66.7%)	0.04 (0.01, 0.42)	0.0013^⁎ Statistically significant at p<0.05
No	12 (33.3%)	11 (91.7%)	1 (8.3%)	REF	

^ ORs generated from Logistic Regressions.

+ P-values generated from Chi square tests or ^Fishers Exact tests if cell count < 5.

- ORs for cells with zero cannot be generated.

# Variable level removed for OR and P-value calculation.

⁎ Statistically significant at p<0.05.

**Table 4:** Odds ratios of cost measure.

Cost measure	Response	Public Insurance	Private Insurance	Odds Ratio (CI)	P-value
Cost-of-Care Conversation Items (N=37)					
Do you believe that incorporating cost-related conversations into pregnancy care is important?^+^	Yes	22 (44.9%)	27 (55.1%)	0.19 (0.05, 0.74)	0.0193^^⁎^
	No	13 (81.6%)	3 (18.8%)	5.32 (1.34, 21.05)	
Did cost conversations occur with:					
provider?	Yes	3 (37.5%)	5 (62.5%)	0.49 (0.10, 2.43)	0.4470^
	No	16 (55.2%)	13 (44.8%)	2.05 (0.41, 10.24)	
partner?	Yes	14 (45.2%)	17 (54.8%)	0.16 (0.12, 1.58)	0.1797^
	No	5 (83.3%)	1 (16.7%)	6.07 (0.63, 58.22)	
family member(s)?	Yes	11 (61.1%)	7 (38.9%)	2.16 (0.58, 8.04)	0.2476
	No	*8 (42.1%)*	*11 (57.9%)*	*0.46 (0.12, 1.72)*	
friend or coworker?	Yes	3 (27.3%)	8 (72.7%)	0.23 (0.05, 1.10)	0.0789^
	No	16 (61.5%)	10 (38.5%)	4.27 (0.91, 19.99)	
someone at clinic (not provider)?	Yes	5 (38.5%)	8 (61.5%)	0.45 (0.11, 1.78)	0.2483
	No	14 (58.3%)	10 (41.7%)	2.24 (0.56, 8.91)	
insurance provider?	Yes	2 (33.3%)	4 (66.7%)	0.41 (0.06, 2.59)	0.4048^
	No	17 (54.8%)	14 (45.2%)	2.43 (0.39, 15.27)	
HR/benefits specialist at work?	Yes	1 (16.7%)	5 (83.3%)	0.14 (0.02, 1.39)	0.0897^
	No	18 (58.1%)	13 (41.9%)	6.92 (0.72, 66.51)	
Cost Tool Implementation Benefits (N=71)					
The cost tool helped me:					
navigate insurance more effectively.	Yes	13 (81.3%)	3 (18.8%)	4.49 (1.15, 17.55)	0.0254^^⁎^
	No	27 (49.1%)	28 (50.9%)	0.02 (0.06, 0.87)	
see the "Big Picture" and link it to the family budget.	Yes	18 (78.3%)	5 (21.7%)	4.25 (1.36, 13.33)	0.0099^⁎^
	No	22 (45.8%)	26 (54.2%)	0.02 (0.08, 0.74)	
feel my financial situation was being considered.	Yes	14 (70.0%)	6 (30.0%)	2.24 (0.74, 6.76)	0.1461
	No	26 (51.0%)	25 (49.0%)	0.45 (0.15, 1.34)	
make the financial tradeoffs needed to get through my pregnancy.	Yes	11 (84.6%)	2 (15.4%)	5.50 (1.12, 27.03)	0.0305^^⁎^
	No	29 (50.0%)	29 (50.0%)	0.18 (0.04, 0.89)	
manage my other life responsibilities.	Yes	23 (65.7%)	12 (34.3%)	2.14 (0.82, 5.58)	0.1163
	No	17 (47.2%)	19 (52.8%)	0.47 (0.18, 1.22)	

^+^ N=65.

^ Fishers Exact Test.

^⁎^ Statistically significant at p < 0.05 for Chi-squared test (or Fisher's exact test if indicated).

## 3 Results

Of the 184 prenatal patient participants who were given a Cost Tool during an OB appointment at any of the three involved clinics, 71 (38.6%) completed follow-up interviews or surveys (study participants). Among the 71 study participants, 34 prenatal patients completed interviews and 37 prenatal patients completed surveys. Overall, most patients were 26-35 years old (57.7%), English-speaking (95.8%), Hispanic/Latino/Spanish (56.3%), and received care at Clinic 2 (56.3%). Pregnancy related, most prenatal patients were low risk (54.9%) and had at least one prior child (56.3%).

Prenatal patients with public insurance were statistically more likely to be Hispanic/Latino/Spanish (OR 6.30, 95% CI 2.23- 17.80; p=0.0003), have received care at Clinic 2 (OR 162.00, CI 16.85 - 999.999; p<0.0001), have received the Cost Tool from by a nurse provider (OR 58.50, CI 5.84 - 586.123, p<0.0001), and be in the youngest age group – 18-25 (OR 8.50, CI 1.01 - 71.70; p=0.006). See Table 3 for more details.

#### 3.1.1 Cost measure: Cost-of-Care conversation items

Prenatal patients with public insurance were less likely to report having cost conversations, specifically with an OB provider (OR 0.49, CI 0.10-2.43; p=0.4470), partner (OR=0.16, CI 0.12-1.58; p=0.1797), friend or coworker (OR=0.23, CI 0.05-1.10; p=0.0789), someone else at the clinic (OR=0.45, CI 0.11, 1.78; p=0.2483), insurance provider (OR=0.41, CI 0.06, 2.59; p=0.4048) or human resources (HR)/benefits specialist at work (OR=0.14, CI 0.02, 1.39; p=0.0897) as compared with prenatal patients with private insurance (Table 4), none of these odds ratios however are statistically significant given the limited sample size, but important signaling nonetheless. Similarly, prenatal patients with public insurance were more likely to report conversations with a family member(s) (OR 2.16, CI 0.58-8.04; p=0.2476) compared with prenatal patients with private insurance. Despite no significant differences in self-reported occurrences of cost-of-care conversations between prenatal patients by insurance status, prenatal patients with public insurance were significantly less likely to report beliefs that incorporating cost-related conversations into pregnancy care is important compared to those with private insurance (OR 0.19, CI 0.05, 0.74; p=0.0193).

#### 3.1.2 Cost measure: Cost Tool implementation benefit items

Implementation of the Cost Tool revealed several statistically significant benefits for prenatal patients with public insurance relative to those with private insurance. Relative to prenatal patients with private insurance, prenatal patients with public insurance were statistically significantly more likely to report the Cost Tool as helping to navigate insurance more effectively (OR 4.49, CI 1.15-17.55; p=0.0254), see the "Big Picture" and link it to the family budget (OR 4.25 CI 1.36-13.33; p=0.0099), and make the financial tradeoffs needed to get through pregnancy (OR 5.50, CI 1.12-27.03; p=0.0305). While not statistically significant, prenatal patients with public insurance were twice as likely to report the Cost Tool as helping to feel their financial situation was being considered (OR 2.24, CI 0.74-6.76; p=0.1461) and manage other life responsibilities (OR 2.14, CI 0.82, 5.58; p=0.1163) as compared with prenatal patients with private insurance.

## 4 Discussion and conclusion

### 4.1 Discussion

The CONTINUE study is a pilot study to gather preliminary data on the potential impact of implementing a cost tool into routine OB care. This study was particularly concerned with addressing whether a cost tool could facilitate cost-of-care conversations and with whom, and more broadly, if there may be benefits of a cost tool in routine OB care.

In respect to whether cost-of-care conversations were occurring in routine OB care with this specific group, low-income prenatal patients, as compared to higher-income prenatal patients were less likely to report having cost-of-care conversations with their OB provider, clinic staff, partner, friend, insurance provider or work associates. On the opposite end, higher-income prenatal patients reported conversations about costs with a broader range of individuals, including OB providers, clinic staff, insurance providers, HR/benefits specialists, friends or coworkers, and partners, as compared to prenatal patients with public insurance. While conversations with insurance providers and HR/benefits specialists can be linked with work status and challenges associated with having private insurance, more personal reasons that may explain differences in conversations with close individuals, like partners and family, should be explored further, especially given no differences in reported presence of partners by insurance type. Most notably, higher- and low-income prenatal patients did not align when it came to welcoming cost-of-care conversations in their routine OB care with higher-income prenatal patients welcoming them and low-income prenatal patients not. This may be due to the stigma associated with being low-income, which research shows low-income patients are more likely to have worse treatment and worse health outcomes because of it [26,27].

As far as reported financial benefits, low-income prenatal patients were more likely to see cost-related benefits as reported by Cost Tool use. Perhaps unsurprisingly, higher-income prenatal patients did not find cost related benefits in the tool, despite welcoming it in OB practice, signaling the Cost Tool may potentially be more beneficial to low-income prenatal patients but could be distributed to anyone, which would be ideal in a clinical setting so as not to target patients due to perceived financial status. The limited sample size made it a challenge to draw widespread conclusions about the financial benefits of a cost tool, but our current data strongly suggests that having an added support in the form of a low-burden tool may be beneficial, even if low-income prenatal patients are not comfortable directly speaking about cost-of-care with their OB provider/s.

#### 4.1.1 Limitations

The CONTINUE study is a pilot sought to gather data on the preliminary benefits of introducing a cost tool in routine care in a varied prenatal patient and OB provider population. Given this was a pilot study, the study size – number of patients, providers, and clinics – had to remain manageable which limited some of results and the ability to achieve statistical significance. Although the results are trending favorably, a larger sample size would render a stronger conclusion. Additionally, given that this is an implementation pilot project, sites and prenatal patients were not randomized which may introduce a selection bias.

### 4.2 Innovation

The CONTINUE study is innovative in that it was able to take a diverse population and gather preliminary data on a tool from multiple clinics with varied OB provider types. It was also able to take semi structured interviews and survey data to combine into key benefit items for quantitative analysis [28]. Given the limitation on cost-of-care conversation research, this study contributes valuable lessons learned about the utility of a cost tool in routine OB care, how to capture data on the effects, and what measures to use.

### 4.3 Conclusion

Based on preliminary findings from the CONTINUE study, there are benefits to cost tools for prenatal patients beyond the potential to facilitate cost-of-care conversations. Prenatal patients with public insurance reported cost-related tool benefits such as helping to navigate costs surrounding burdensome OB care plans, making financial tradeoffs, and linking costs associated with pregnancy to the family budget, all of which are essential for care adherence. Given this, the Cost Tool has value to all prenatal patients, even if publicly and privately insured prenatal patients are not deriving the same benefit from tool use. This study called into question that perhaps the goal of promoting cost-of-care conversations is not the right goal for low-income women, but instead the goal should be giving low-income women the foresight to allow them to take the actions they prefer and need, whether a cost-of-care conversation happens.

An important distinction needs to be made between the presence of cost-of-care conversations and the tangible cost-related benefits it promotes. While low-income prenatal patients reported less cost-of-care conversations overall, they also reported cost-related benefits that helped them navigate the financial burden of their OB care plan. This is particularly important in this population, given low-income women are more likely to postpone and forego care [20]. More notably, low-income patients were less likely to report that cost-of-care conversations with their OB providers as desirable in pregnancy care. The reason for this should be explored further, especially given research indicating that these types of conversations can optimize care. Previous research has suggested that low-income patients do not want to feel “singled out” and have care predicated on their perceived income level, which may be why low-income patients are less likely to report cost-of-care conversations as desirable.

## Funding

This study was funded by the 10.13039/100000867Robert Wood Johnson Foundation (grant # 77290). The funding body had no input on the design of the study and collection, analysis, and interpretation of data and in writing the manuscript. The authors declare that they have no competing interests.

## Declaration of Competing Interest

The authors declare that they have no known competing financial interests or personal relationships that could have appeared to influence the work reported in this paper.

## References

[1] Meluch A.L., Oglesby W.H. (2015). Physician–patient communication regarding patients’ healthcare costs in the US: A systematic review of the literature. J Commun Healthc.

[2] Piette J.D., Heisler M., Wagner T.H. (2004 Sep 13). Cost-related medication underuse: Do patients with chronic illnesses tell their doctors?. Arch Intern Med.

[3] Barkil-Oteo A., Stern D.A., Arbuckle M.R. (2014). Addressing the cost of health care from the front lines of psychiatry. JAMA Psychiatry.

[4] Harrington N.G., Scott A.M., Spencer E.A. (2020 Aug). Working toward evidence-based guidelines for cost-of-care conversations between patients and physicians: A systematic review of the literature. Soc Sci Med.

[5] Shih Y.T., Chiun-Ru C. (2017 May 15). A review of cost communication in oncology: Patient attitude, clinician acceptance, and outcome assessment. Cancer..

[6] Zafar S.Y., Chino F., Ubel P.A., Rushing C., Samsa G., Altomare I. (2015 Sep). The utility of cost discussions between patients with cancer and oncologists. Am J Manag Care.

[7] Patel M.R., Wheeler J.R. (2014 Dec). Physician–patient communication on cost and affordability in asthma care. Who wants to talk about it and who is actually doing it. Ann Am Thorac Soc.

[8] Bestvina C.M., Zullig L.L., Rushing C., Chino F., Samsa G.P., Altomare I. (2014 May 1). Patient-oncologist cost communication, financial distress, and medication adherence. J Oncol Pract.

[9] Perez S.L., Weissman A., Read S., Smith C.D., Colello L., Peter D. (2019 May 7). U.S. internists’ perspectives on discussing cost of care with patients: Structured interviews and a survey. Ann Intern Med.

[10] Wollins D.S., Zafar S.Y. (2016 Oct). A touchy subject: Can physicians improve value by discussing costs and clinical benefits with patients?. Oncologist..

[11] Hunter W.G., Hesson A., Davis J.K., Kirby C., Williamson L.D., Barnett J.A. (2016 Mar). Patient-physician discussions about costs: Definitions and impact on cost conversation incidence estimates. BMC Health Serv Res.

[12] Hunter W.G., Zhang C.Z., Hesson A., Davis J.K., Kirby C., Williamson L.D. (2016 Oct). What strategies do physicians and patients discuss to reduce out-of-pocket costs? Analysis of cost-saving strategies in 1,755 outpatient clinic visits. Med Decis Mak.

[13] Dine C.J., Masi D., Smith C.D. (2019 May 7). Tools to help overcome barriers to cost-of-care conversations. Ann Intern Med.

[14] Alexander G.C., Casalino L.P., Tseng C.W., McFadden D., Meltzer D.O. (2004 Aug). Barriers to patient-physician communication about out-of-pocket costs. J Gen Intern Med.

[15] Fostering Productive Health Care Cost Conversations (2023). Sharing Lessons Learned and Best Practices. acpjournals.org. https://www.acpjournals.org/toc/aim/170/9_Supplement.

[16] Brick D.J., Scherr K.A., Ubel P.A. (2019 Jan). The impact of cost conversations on the patient-physician relationship. Health Commun.

[17] Rosenfeld J. (2023). Navigating cost-of-care conversations with patients. medicaleconomics.com. https://www.medicaleconomics.com/view/navigating-cost-care-conversations-patients.

[18] Bengiamin M.I., Capitman J.A., Ruwe M.B. (2010). Disparities in initiation and adherence to prenatal care: impact of insurance, race-ethnicity and nativity. Matern Child Health J.

[19] Seervai Shanoor (Feb. 6, 2019). Practicing Medicine in Rural America: Listening to Primary Care Physicians. Commonwealth Fund.

[20] Right Place (January 2017). https://altarum.org/sites/default/files/uploaded-publication-iles/USE_RPRT_ConsumerPerspectives_Final.pdf.

[21] The American College of Obstetricians and Gynecologists (ACOG) & American Academy of Pediatrics (AAP) (2017 Sept). Guidelines for Perinatal Care 8^th^ Ed. https://www.acog.org/clinical-information/physician-faqs/-/media/3a22e153b67446a6b31fb051e469187c.ashx.

[22] Erwin K., Fitzpatrick V., Norell S. Gilliam, M. (2019). Development of a Framework and Tool to Facilitate Cost-of-Care Conversations With Patients During Prenatal Care. Ann Intern Med.

[23] Ngo E., Truong M.B.T., Nordeng H. (2020). Use of decision support tools to empower pregnant women: systematic review. J Med Internet Res.

[24] Akkerman S.F., Bakker A. (2011). Boundary crossing and boundary objects. Rev Educ Res.

[25] Melville-Richards L., Rycroft-Malone J., Burton C., Wilkinson J. (2020). Making authentic: exploring boundary objects and bricolage in knowledge mobilisation through National Health Service-university partnerships. Evid Policy.

[26] Sheppard V.B., Zambrana R.E., O’Malley A.S. (2004). Providing health care to low-income women: A matter of trust. Fam Pract.

[27] Tandon S.D., Parillo K.M., Keefer M. (2005 Dec). Hispanic women’s perceptions of patient-centeredness during prenatal care: a mixed-method study. Birth..

[28] Rivelli A., Fitzpatrick V., Shields K. (2023).

